# A Protective Monoclonal Antibody Targets a Site of Vulnerability on the Surface of Rift Valley Fever Virus

**DOI:** 10.1016/j.celrep.2018.12.001

**Published:** 2018-12-26

**Authors:** Elizabeth R. Allen, Stefanie A. Krumm, Jayna Raghwani, Steinar Halldorsson, Angela Elliott, Victoria A. Graham, Elina Koudriakova, Karl Harlos, Daniel Wright, George M. Warimwe, Benjamin Brennan, Juha T. Huiskonen, Stuart D. Dowall, Richard M. Elliott, Oliver G. Pybus, Dennis R. Burton, Roger Hewson, Katie J. Doores, Thomas A. Bowden

**Affiliations:** 1 Division of Structural Biology, Wellcome Centre for Human Genetics, University of Oxford, Roosevelt Drive, Oxford OX3 7BN, UK; 2 Kings College London, Department of Infectious Diseases, 2nd Floor, Borough Wing, Guy’s Hospital, Great Maze Pond, London SE1 9RT, UK; 3 Big Data Institute, Li Ka Shing Centre for Health Information and Discovery, Nuffield Department of Medicine, University of Oxford, Old Road, Oxford OX3 7LF, UK; 4 MRC-University of Glasgow Centre for Virus Research, Institute of Infection, Immunity and Inflammation, College of Medical, Veterinary and Life Sciences, University of Glasgow, 464 Bearsden Road, Glasgow G61 1QH, UK; 5 National Infection Service, Virology & Pathogenesis, Public Health England, Porton Down, Salisbury, SP4 0JG Wiltshire, UK; 6 The Jenner Institute, University of Oxford, Oxford OX3 7DQ, UK; 7 Centre for Tropical Medicine and Global Health, University of Oxford, Oxford OX3 7FZ, UK; 8 Kenya Medical Research Institute (KEMRI)-Wellcome Trust Research Programme, Kilifi, Kenya; 9 Department of Zoology, University of Oxford, South Parks Road, Oxford, UK; 10 Department of Immunology and Microbiology, The Scripps Research Institute, La Jolla, CA 92037, USA; 11 Ragon Institute of MGH, Harvard, and MIT, Cambridge, MA 02139, USA

**Keywords:** phlebovirus, Rift Valley fever virus, antibody, structure, bunyavirus, virus-host interactions, immune response, vaccine, antiviral, neutralization

## Abstract

The Gn subcomponent of the Gn-Gc assembly that envelopes the human and animal pathogen, Rift Valley fever virus (RVFV), is a primary target of the neutralizing antibody response. To better understand the molecular basis for immune recognition, we raised a class of neutralizing monoclonal antibodies (nAbs) against RVFV Gn, which exhibited protective efficacy in a mouse infection model. Structural characterization revealed that these nAbs were directed to the membrane-distal domain of RVFV Gn and likely prevented virus entry into a host cell by blocking fusogenic rearrangements of the Gn-Gc lattice. Genome sequence analysis confirmed that this region of the RVFV Gn-Gc assembly was under selective pressure and constituted a site of vulnerability on the virion surface. These data provide a blueprint for the rational design of immunotherapeutics and vaccines capable of preventing RVFV infection and a model for understanding Ab-mediated neutralization of bunyaviruses more generally.

## Highlights

• The Gn glycoprotein of Rift Valley fever virus elicits potent neutralizing antibodies
• Derived a class of monoclonal antibodies that protects in an animal model
• A distinct region on RVFV Gn constitutes a key site of vulnerability
• Antibodies are predicted to prevent exposure of viral fusion loops

## Graphical Abstract

**Figure:**
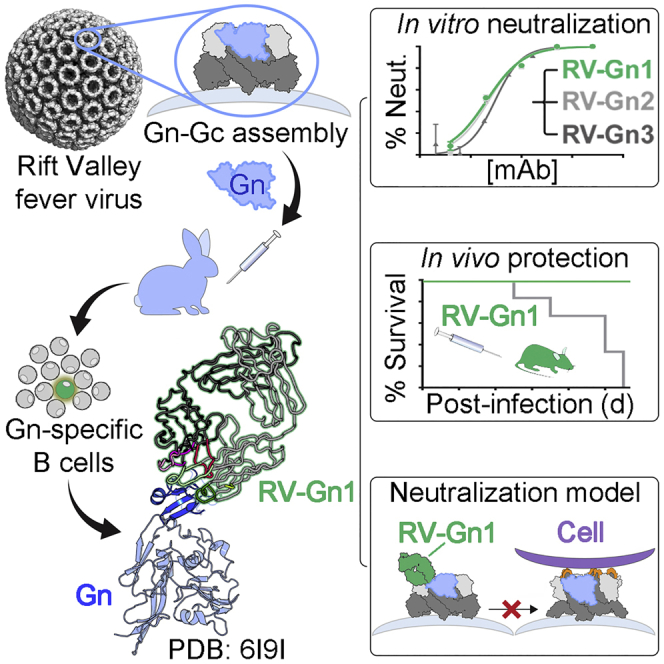

## Teaser

Allen et al. reveal a molecular basis of antibody-mediated neutralization of Rift Valley fever virus, an important human and animal pathogen. They isolate and demonstrate the protective efficacy of a monoclonal antibody in a murine model of virus infection, providing a blueprint for rational therapeutic and vaccine design.

## Introduction

First reported in 1931 (Daubney et al., 1931), Rift Valley fever virus (RVFV) is an arbovirus endemic to Africa and the Arabian peninsula that causes recurrent epidemics and epizootics. RVFV is of both agricultural and biomedical importance, as infection of livestock results in high incidences of neonatal mortality and zoonosis; human disease ranges from mild self-limiting febrile illness to severe disease characterized by hemorrhagic diatheses, encephalitis, and ocular pathologies (Al-Hazmi et al., 2003, Mohamed et al., 2010, Sow et al., 2014). Human populations throughout East Africa are at high risk for RVFV infection, with seroprevalence reported to exceed 8% in communities located near water reservoirs that support mosquito populations (Pourrut et al., 2010). No licensed antivirals or vaccines for RVFV are currently available, although a number of vaccine candidates are in development (Dungu et al., 2018, Faburay et al., 2017).

The genetically diverse group of viruses within the genus *Phlebovirus*, family *Phenuiviridae*, currently contains ten species (Adams et al., 2017). Like all known phleboviruses, RVFV is enveloped and contains a single-stranded, negative- or ambi-sense RNA genome that is divided into three segments: S, M, and L. The M segment encodes the glycoprotein precursor, which is processed into two membrane-anchored glycoproteins, Gn and Gc (Gerrard and Nichol, 2007). While the phleboviral Gc forms a class II fusion architecture observed in a number of viral families (Dessau and Modis, 2013, Guardado-Calvo et al., 2017, Halldorsson et al., 2016, Vaney and Rey, 2011, Zhu et al., 2017), the multi-domain phleboviral Gn is structurally distinct and exhibits partial secondary structure similarity with the Gn of hantaviruses and the E1 of alphaviruses (Guardado-Calvo and Rey, 2017, Halldorsson et al., 2018, Li et al., 2016, Rissanen et al., 2017, Voss et al., 2010, Wu et al., 2017). In contrast to the phleboviral Gc, which is structurally well conserved, studies of RVFV Gn and SFTSV Gn have revealed that the phleboviral Gn maintains a low level of structural conservation across the family (approximately 3 Å root-mean-square deviation [RMSD]) (Wu et al., 2017).

Heterodimers of RVFV Gn and Gc form pentameric and hexameric assemblies on the virion surface in an icosahedral *T* = 12 organization (Freiberg et al., 2008, Halldorsson et al., 2018, Sherman et al., 2009). Structural studies localize the N-terminal domains of RVFV Gn to the membrane-distal region and have proposed that it functions as a molecular shield that protects against premature fusogenic rearrangements of the cognate Gc (Halldorsson et al., 2018). Host-cell entry of RVFV is instigated by attachment of Gn-Gc-associated oligomannose-type glycans to the C-type lectin DC-SIGN (Crispin et al., 2014, Lozach et al., 2011, Phoenix et al., 2016). Following caveolae-mediated endocytic uptake, displacement of the Gn is expected to expose the Gc and allow fusion of the virion and cellular membranes in a histidine-triggered pH-dependent process (de Boer et al., 2012, Halldorsson et al., 2016, Harmon et al., 2012, Lozach et al., 2010).

Recovery from RVFV infection is associated with the development of high titers of neutralizing antibodies, which convey long-lasting protection against further infection (Bird and Nichol, 2012, Madani et al., 2003, Sabin and Blumberg, 1947). Although antibodies are elicited against structural and non-structural protein components of the phlebovirus during infection (Boshra et al., 2011, Brown et al., 1957, Fernandez et al., 2012, Zaki et al., 2006), neutralizing monoclonal antibodies (nAbs) are predominantly raised against the Gn and Gc glycoproteins, revealing them to be important targets for vaccine and antiviral design (Faburay et al., 2017).

We sought to investigate the molecular basis of RVFV neutralization by the humoral immune response. Using recombinant RVFV Gn as an immunogen, we isolated a class of Gn-specific rabbit monoclonal nAbs, which protect against RVFV challenge in a murine infection model. X-ray crystallographic analysis reveals that these nAbs target the membrane-distal head region of RVFV Gn. These data provide a molecular rationale for understanding Ab-mediated targeting of RVFV and establish a domain on RVFV Gn as a region of immune vulnerability on the phleboviral surface.

## Results

### RVFV Gn Glycoprotein Elicits a Protective Neutralizing Ab Response

**Figure 1:**
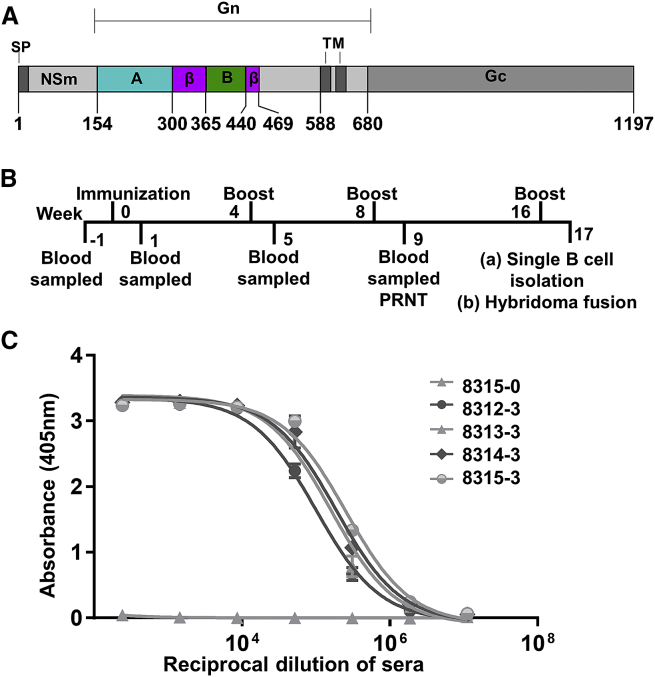
Immunization with Recombinant RVFV Gn Elicits a Robust IgG Response (A) Domain organization of the RVFV M segment. The full-length ectodomain of RVFV Gn was used for immunization. The RVFV Gn construct used for crystallization is colored according to structural identified domains: domain A (cyan), β-ribbon domain (β, magenta), and domain A (green). The signal peptide (SP), NSm protein, transmembrane region (TM), and Gc glycoprotein are annotated. (B) Timeline of rabbit immunization experiments. Rabbits were immunized with recombinant RVFV Gn and boosted at 4 week intervals. Seven days following the second boost, RVFV Gn binding and neutralization titers were measured. A splenectomy was performed 7 days following the third boost. mAbs were derived by hybridoma fusion from splenocytes and antigen-specific single B cell sorting of PBMCs. (C) ELISA measuring the titers of IgG specific to RVFV Gn for each rabbit (rabbits 8312–8315) following the third boost. Sample 8315-0 is a pre-immunization sera control derived from rabbit 8315. See also Figure S1. Error bars represent the range of the value for experiments performed in duplicate (not shown when smaller than symbol size).

As neutralizing Abs raised during infection and immunization have been shown to target the phleboviral Gn (Faburay et al., 2017), we hypothesized that recombinantly expressed RVFV Gn would constitute an effective immunogen and could be used to elicit nAbs. We immunized four New Zealand white rabbits with RVFV Gn and, following the second boost, observed a potent neutralizing IgG response against the glycoprotein (Figures 1A–1C; Table S1).

**Figure 2:**
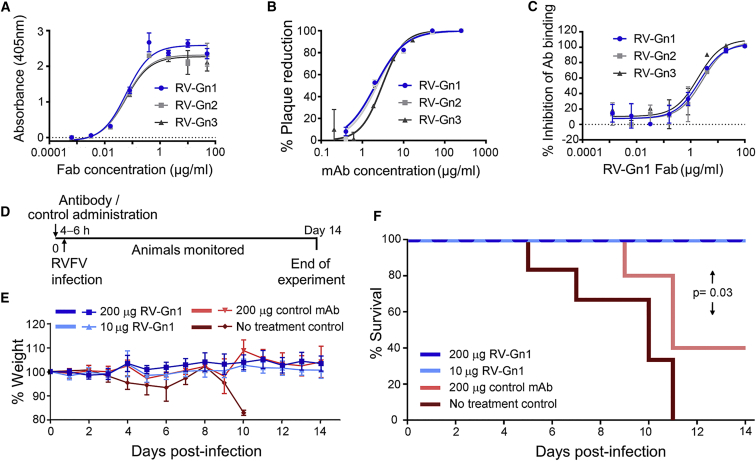
High-Affinity mAbs RV-Gn1–3 Are Neutralizing, Target-Overlapping Epitopes on RVFV Gn and Protect against RVFV Infection in a Mouse Model (A) ELISA analysis of mAbs RV-Gn1, RV-Gn2, and RV-Gn3 titrated with RVFV Gn. Experiments were performed in duplicate and repeated three times. A representative dataset is shown. (B) RVFV-Gn1, RVFV-Gn2, and RVFV-Gn3 neutralize RVFV in a plaque reduction neutralization test. Data are shown as percentage reduction in plaques compared with the mAb control, and a non-linear regression model is fitted using GraphPad Prism. Experiments were performed in duplicate. (C) Competition ELISA analysis: overlap of RV-Gn1–3 epitopes were determined by binding of full-length IgG to RVFV Gn in the presence of RV-Gn1 Fab. Experiments were performed in duplicate and repeated three times. A representative dataset is shown. Error bars in (A)–(C) represent the range of the value for experiments performed (not shown when smaller than symbol size). (D) Timeline of mouse protection experiments. Mice (n = 6) were given RV-Gn1 or control antibody 8 hr prior to infection with RVFV. Clinical signs of infection and survival were recorded over 14 days. (E) Weight loss was recorded over the time course of the experiment. Error bars represent the SEM (not shown when smaller than symbol size). (F) Efficacy of antibody protection was assessed by a survival analysis. A Kaplan-Meier log rank test was performed; p = 0.03 between RV-Gn1-treated and mAb control, p = 0.008 between RV-Gn1-treated and no-treatment control, p = 0.08 between mAb control and no-treatment control. See also Figure S2 and Tables S1 and S2.

We sought to understand the molecular mechanism underlying the response generated by immunization. We isolated approximately 100 hybridomas and screened their supernatants for RVFV neutralization potency. This analysis revealed that approximately one-third of the hybridomas were able to neutralize RVFV *in vitro*, demonstrating that our RVFV Gn construct is an effective immunogen. From the isolated hybridomas, we recovered sequences for the heavy- and light-chain pairings for two strongly binding monoclonal antibodies (mAbs), termed RV-Gn1 and RV-Gn2. We further isolated an additional strongly binding mAb, termed RV-Gn3, by antigen-specific single B cell sorting of peripheral blood mononuclear cells (PBMCs) (Figures 2A, S1, and S2). A plaque reduction neutralization test (PRNT) demonstrated that each of the three mAbs neutralized RVFV with half-maximal inhibitory concentration (IC_50_) values ranging from 2.1 to 3.0 μg/mL (Figure 2B).

RV-Gn1, RV-Gn2, and RV-Gn3 exhibit high levels of sequence conservation in the complementarity-determining regions (CDRs) of each respective fragment antigen binding (Fab) region (Figure S2). Furthermore, analysis of germline V, J, and D segments suggests that these mAbs are likely clonally related, with 8%–12% mutation from germline (combined V and J) for both the heavy and light chains (Table S2; Lefranc et al., 1999). An ELISA-based competition assay indicates that these closely related mAbs target a common epitope (Figure 2C). The commonalities in sequence, binding affinity, and epitope suggest that RV-Gn1, RV-Gn2, and RV-Gn3 can be categorized as a single class of nAb.

To determine whether this class of anti-RVFV Gn nAb also protects against disease, female BALB/c mice were intravenously administered with 10 or 200 μg of RV-Gn1 8 hr prior to challenge with 20 plaque-forming units of RVFV (strain ZH501). Mice were monitored for disease 14 days post-infection. While none of the untreated mice survived RVFV challenge, a 40% survival rate was observed for the IgG isotype control group, and 100% survival rate was observed for the RV-Gn1-treated mice, irrespective of the RV-Gn1 dose used (Figures 2D–2F). A significant difference was observed between the RV-Gn1-treated mice and the isotype controls (p = 0.03, Kaplan Meier with log rank). Although we observed increased survival in the isotype control group compared with the no-treatment control group, this was not significant (p = 0.08). We suggest that the increased survival rate in the IgG control group may either reflect a feature of the animal system used, as has been observed in other protection studies against emerging viruses (Zhao et al., 2017), or be a non-specific effect of treatment with IgG. Nevertheless, the 100% survival of mice following treatment with RV-Gn1 indicates the therapeutic potential of anti-RVFV mAbs, which target the virus surface.

### RV-Gn nAbs Target Domain B of RVFV Gn

**Figure 3:**
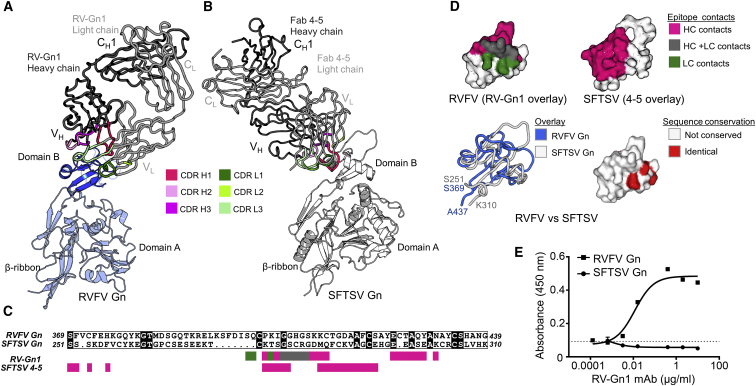
Structural Basis for RV-Gn1-Mediated Neutralization of RVFV (A) Crystal structure of recombinantly expressed monomeric RVFV Gn in complex with the Fab fragment of RV-Gn1. Domain B of RVFV Gn is shown as a blue cartoon. The β-ribbon domain and domain A (light blue cartoon) were cleaved during crystallogenesis and are modeled by superposition with the previously reported crystal structure of unliganded RVFV Gn (PDB: 6F8P) (Halldorsson et al., 2018). V_H_, V_L_, C_H_1, and C_L_ denote the antibody variable heavy, variable light, constant heavy 1, and constant light chain domains, respectively. Complementarity-determining regions (CDRs) are colored shades of pink (heavy chain) and green (light chain). (B) Crystal structure of SFTSV Gn in complex with Fab 4-5 (PDB: 5Y11) (Wu et al., 2017), as presented in (A). (C) Sequence alignment of SFTSV Gn (residues 251–310) and RVFV Gn (residues 370–439) (calculated by Clustal Omega [Sievers et al., 2011] and plotted with ESPript [Robert and Gouet, 2014]). Black squares around residues indicate identity. Colored squares below the sequence mark residues forming contacts with either RV-Gn1 (upper) or SFTSV Gn 4-5 (lower). Residues contacted by the heavy (V_H_) chain and light chain (V_L_) are shown in pink and green, respectively. Residues contacted by both chains are shown in gray. (D) Footprints of RV-Gn1 and Fab 4-5 plotted onto RVFV Gn domain B and SFTSV domain B, respectively, reveals contrasting modes of antigen recognition. Upper: antibody footprints mapped onto domain B of RVFV Gn (RV-Gn1, left) and SFTSV Gn (4-5, right). Structures are shown in surface representation with residues colored as annotated in (C) Lower left: structure overlay of domain B from RVFV Gn (blue) and SFTSV Gn (white) revealed a 1.8 Å root-mean-square-deviation (RMSD). Lower right: structure-based mapping of sequence conservation between RVFV Gn and SFTSV Gn. RVFV Gn is shown in surface representation with identical residues colored red and non-conserved residues colored white. (E) ELISA analysis reveals that RV-Gn1 does not bind SFTSV Gn. Wells were coated with SFTSV Gn or RVFV Gn and titrated with RV-Gn1 (performed in triplicate). The dotted line indicates background binding observed in the negative control. Error bars represent the SEM (not shown when smaller than symbol size). See also Figure S3 and Table S3.

Previous structural analyses of RVFV Gn have revealed a triangular organization composed of three domains: domain A (residues 154–300), domain B (residues 366–439), and a β-ribbon domain (residues 301–365 and 440–469) (Halldorsson et al., 2018, Wu et al., 2017). We sought to ascertain the epitopes targeted on RVFV Gn by our class of protective nAbs (RV-Gn1–3) and the molecular basis for mAb-mediated neutralization. Following complexation of recombinantly derived RVFV Gn monomer with the Fab region of RV-Gn1, which suggested a 1:1 RVFV Gn-Fab stoichiometry (Figure S3), we crystallized and determined the structure of the complex to 1.98 Å resolution (Figure 3A; Table S3).

The two complexes of RVFV Gn-Fab RV-Gn1 in the asymmetric unit exhibit near-identical RVFV Gn binding modes, with both binding to the apex of domain B in the Gn (0.5 Å RMSD). Interestingly, only residues constituting domain B were resolved in our crystallographic dataset (residue numbers 370–379 and 397–437). Electron density corresponding to both domain A and the β-ribbon domain of RVFV Gn was not visible and could not be sterically accommodated in the RVFV Gn-Fab RV-Gn1 crystal, indicating that these regions were likely cleaved during crystallogenesis.

Although CDR loops from both the heavy and light chains of RV-Gn1 contribute to the approximately 800 Å^2^ interface, the heavy chain dominates and forms 12 of 15 hydrogen bonds in the protein-protein interaction. Loops 405–431 and 423–431 of domain B in RVFV Gn play a central role in the interaction and maintain a conformation observed in unliganded structures of RVFV Gn, consistent with the high level of structural similarity between nAb-bound and nAb-free RVFV Gn structures (1.1 Å RMSD). Furthermore, we note that RV-Gn1 residues that form the paratope are highly conserved with RV-Gn2 and RV-Gn3 (Figure S2), suggesting that all of our identified Abs use a highly similar mode of antigen recognition.

We note that a neutralizing Ab (MAb 4-5) has also been structurally characterized in complex with the Gn of SFTSV (Wu et al., 2017), a genetically, antigenically, and structurally distal relative of RVFV that shares only 24% sequence identity in Gn (Figures 3B–3D; Cui et al., 2015, Yu et al., 2011). Interestingly, although RV-Gn1 and MAb 4-5 use distinct modes of binding with dissimilar CDR loop usage, contact antigenically distinctive surfaces, and do not cross-react with Gns by ELISA (Figure 3E), their epitopes localize to domain B of their respective phleboviral Gn (Figure 3), suggesting that the Ab-mediated targeting of this portion of the molecule may be a universal feature of immune responses to phleboviruses and therefore a common domain for immunogen design strategies.

**Figure 4:**
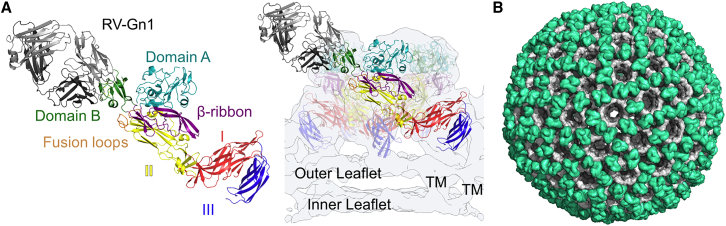
RVFV-Gn1 Recognizes the Membrane-Distal Surface of the RVFV Gn-Gc Assembly (A) Modeling of RV-Gn1 Fab onto RVFV Gn-Gc heterodimers (PDB: 6F9F) reveals an epitope proximal to Gc-resident hydrophobic fusion loops. RVFV Gc is colored according to domain, with domain I in red, domain II in yellow, and domain III in blue. Right: modeling of RV-Gn1 onto the pentameric assembly of RVFV Gn-Gc heterodimers (PDB: 6F9F). Density corresponding to the cryoelectron microscopy-derived reconstruction of the RVFV virion (EMD: 4201) is shown as a transparent surface, with transmembrane regions and leaflets of the virion lipid-bilayer membrane annotated. (B) The RV-Gn1 epitope is sterically accessible to full occupancy across the RVFV virion. RV-Gn1 is shown in green, and the Gn-Gc complex assembly of RVFV (EMD: 4197) is shown in gray.

To map the location of the RV-Gn1 epitope in the context of the mature RVFV virion, we superimposed the Gn component of our crystallized Fab-Gn complex onto a reported model of assembled RVFV Gn-Gc (Figures 4A and 4B; Halldorsson et al., 2018). This model of the entire RVFV virion reveals that Fab RV-Gn1 targets the membrane-distal region of the glycoprotein assembly in a binding mode that extends the Fab perpendicularly from the virion surface (Figure 4A). Although a 1:1 Fab-to-RVFV Gn stoichiometry could be achieved across the entire virion (Figure 4B), given the size of the corresponding Fc region, it is unlikely that this level of occupancy is required to sterically preclude virus-host interactions.

### Immune-Accessible Domain B of RVFV Gn Is under Selective Pressure

Our structural analysis provides a molecular rationale for the way in which RV-Gn1–3 targets and neutralizes RVFV (Figures 3A and 4). We sought to investigate the evolutionary selective pressures acting on RVFV Gn and to assess whether the identified RV-Gn1 epitope may be targeted by Abs developed during natural infection. Using 98 publicly available gene sequences of RVFV Gn-Gc sampled between 1951 and 2010, we performed a comparative analysis of non-synonymous to synonymous nucleotide substitution ratios (dN/dS) in order to identify regions of the Gn-Gc complex assembly that exhibit greater positive selection for amino acid change (i.e., higher dN/dS ratios).

**Figure 5:**
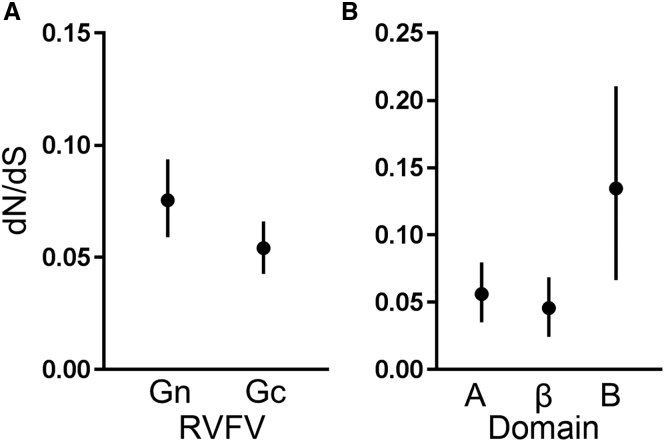
Sequence Diversification across RVFV Gn and Gc Glycoproteins Site-wise dN/dS analysis of the RVFV Gn and Gc. Comparison of dN/dS values between (A) Gn and Gc and (B) structural domains of RVFV Gn, as defined in Figure 1A. Error bars show the 95% highest posterior density intervals, and circles indicate mean values.

In this analysis, we observe that the absolute rate of nucleotide substitution for RVFV Gn-Gc (∼3 × 10^−4^ substitutions/site/year) is an order of magnitude lower than that of the envelope glycoproteins from fast-evolving viruses such as HIV-1 (Env; 2–5 × 10^−3^ substitutions/site/year) (Patiño-Galindo and González-Candelas, 2017), HCV (E1/E2; 1–3 × 10^−3^ substitutions/site/year) (Gray et al., 2011), and seasonal influenza (HA; 5.7 × 10^−3^ substitutions/site/year) (Rambaut et al., 2008). Interestingly, division of the RVFV M segment into Gn and Gc glycoprotein components reveals that the Gn (dN/dS = 0.075) exhibits a marginally higher mean dN/dS than the cognate Gc (dN/dS = 0.054) (Figure 5A). Although this observation is consistent with a higher selective pressure on Gn, it provides limited insight, because the difference in dN/dS ratios is small and because substitution rates are averaged across all codons within the protein. Because most residues are under strong negative selection, such averaging can mask strong heterogeneity in positive selection pressure among subgenic regions.

We therefore subdivided RVFV Gn into its structurally observed A, B, and β-ribbon domains and calculated dN/dS separately for each domain. This additional analysis indicates a significantly higher dN/dS ratio (0.135) for domain B (Figure 5B). This result supports the hypothesis that Gn domain B of the RVFV Gn-Gc complex is subject to the greatest level of immune-mediated selective pressure. Furthermore, the previous observation that this region of SFTSV Gn is also targeted by nAbs (Figures 3B and 3D; Wu et al., 2017) suggests that domain B of the Gn may be an immunodominant region among phleboviruses more generally. We note that amino acid diversification of this region, although slow, may also be facilitated by a greater level of structural plasticity in RVFV Gn domain B compared with other domains, as has been previously inferred in structural studies of the entire RVFV virion (Halldorsson et al., 2018).

## Discussion

The assembly of Gn and Gc glycoproteins that encapsulate the surface of RVFV constitutes a primary target of the nAb response generated during both natural infection and immunization (Faburay et al., 2017). Immunization of rabbits with the monomeric N-terminal ectodomain of RVFV Gn was sufficient to elicit a highly neutralizing Ab response in rabbits (Figure 1; Table S1). These data confirm the Gn glycoprotein as a desirable component of any humoral-based vaccine against RVFV and provide a rational platform for guiding immunogen design efforts for at risk human and animal populations.

**Figure 6:**
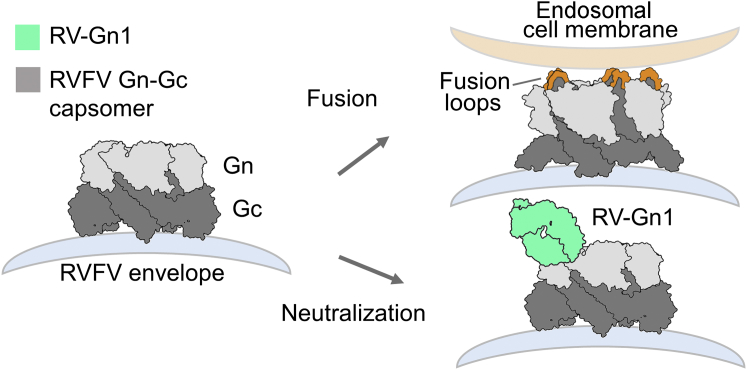
A Schematic Model of RV-Gn1-Mediated Neutralization Domain B-targeting nAbs sterically impede the exposure of Gc-resident hydrophobic fusion loops. In the absence of RV-Gn1, fusion loops encoded in the Gc glycoprotein are able to extend and insert into endosomal membranes of the host cell, facilitating fusion.

We further derived RVFV Gn-specific Abs (RV-Gn1–3) using two complementary techniques: hybridoma fusion from spleen and antigen-specific single B cell sorting of PBMCs. Interestingly, these mAbs (RV-Gn1–3) appear to constitute a single class of nAb, as they recognize an overlapping RVFV Gn epitope and likely originate from a single germline (Figure S2). Structural elucidation of RV-Gn1 in complex with RVFV Gn reveals an 800 Å epitope on domain B of RVFV Gn and provides a molecular basis for immune-mediated neutralization (Figure 3A). Interestingly, this region of the Gn has been shown to shield the Gc against premature fusogenic rearrangements and shifts position upon exposure of RVFV to acidic pH (Halldorsson et al., 2018). Given the close proximity of the RV-Gn1 epitope to the Gn-Gc interface, it is likely that it functions to sterically impede rearrangements to the glycoprotein surface of RVFV, preventing exposure of the RVFV Gc-resident hydrophobic fusion loops in the endosomal membranes following virion uptake into the host cell (Figure 6). However, we cannot preclude the possibility that RV-Gn1 may also disrupt attachment of DC-SIGN to some of the heterogeneously distributed oligomannose-type glycans presented on RVFV Gn and Gc (Lozach et al., 2011, Phoenix et al., 2016). It will be of interest to assess if this immunogen-elicited mode of neutralization is reciprocated during natural human and animal infection.

Despite the genetic, antigenic, and structural differences between the Gn of RVFV and SFTSV (Figures 3C and 3D), the isolation of a nAb from a human SFTSV survivor that targets a similarly localized neutralizing epitope on domain B of SFTSV Gn (Figure 3B) supports a common mechanism of neutralization and is suggestive that this domain of the Gn may constitute a site of vulnerability for phleboviruses more broadly. This hypothesis is supported by our evolutionary analysis of RVFV Gn-Gc, which reveals that amino acid diversification of domain B is greater than that of the rest of the Gn-Gc assembly and is likely subjected to a greater level of immune-mediated selective pressure compared with other regions (Figure 5). Interestingly, however, the overall rate of diversification of evolution in domain B (and Gn-Gc) is still limited compared with other well-characterized viruses, including HIV-1 (Patiño-Galindo and González-Candelas, 2017), influenza virus (Rambaut et al., 2008), and HCV (Gray et al., 2011), suggesting that a multivalent vaccine, such as that being developed against influenza virus, may protect against immune escape in this region (Thompson et al., 2018). Given the analogous putative role of the Gn glycoprotein in protecting the Gc-resident fusion loops in hantaviruses (Li et al., 2016), it will be of interest to determine whether this mechanism of neutralization will also be observed among other families within the *Bunyavirales*.

The existence of an immunodominant region on the surface of phleboviruses has important implications for the development of glycoprotein therapeutics. For example, a reverse vaccinology approach (Burton, 2017) that focuses on domain B of the Gn may benefit immunogen design efforts for pathogenic phleboviruses for which there are no established vaccines, such as SFTSV or Toscana virus. Indeed, the development of such a protein subunit vaccine may also provide an attractive alternative to live-attenuated or inactivated RVFV vaccines, such as MP-12 and TSI-GSD-200, respectively (Dungu et al., 2018), by immunofocusing the Ab response to a vulnerable region of the virion.

The development of antiviral biologics has proved to be a highly effective strategy to protect against emerging viral pathogens (Jin et al., 2017). Our study provides an initial benchmark for the derivation of highly potent therapeutic Abs from immunization or convalescent sera that can be used for the treatment or prevention of RVFV infection. Demonstration of the *in vivo* protective efficacy of our mAb, RV-Gn1, in animals highlights the potential utility of RVFV-specific nAbs as prophylactics. By analogy with mAb cocktails developed against EBOV GP (Qiu et al., 2012), we anticipate that the identification of nAbs specific to other spatially distinct epitopes on the surface of the RVFV Gn-Gc complex assembly will be an important consideration for the development of synergetic, non-competing combinations of anti-RVFV mAbs.

## STAR★Methods

### Key Resources Table

**Table:** 

REAGENT or RESOURCE	SOURCE	IDENTIFIER
**Antibodies**		

Anti-RVFV Gn Rv-Gn1-3	This paper	N/A
goat anti-rabbit IgG F(ab’)_2_, AP conjugate	Invitrogen	cat# 31343
anti-HIV monoclonal Ab PG9	Walker et al., 2009, Walker et al., 2011	N/A
anti-CD3-FITC	Santa Cruz Biotechnology	cat# sc-20047 FITC
anti-IgM-PE	Southern Biotech	cat# 4020-09
anti-IgG-PerCP-Cy5.5	Santa Cruz Biotechnology	cat# sc-45110
anti-HIS-APC	Abcam	cat# ab49936; RRID AB_867459
goat anti-mouse IgG Fc, biotin conjugate	Invitrogen	cat# 31805

**Bacterial and Virus Strains**		

Rift Valley fever virus	PHE	strain ZH501

**Chemicals, Peptides, and Recombinant Proteins**		

RVFV Gn	Halldorsson et al., 2018	N/A
RVFV Gn (crystallization construct)	This paper	N/A
SFTSV Gn	Wu et al., 2017	N/A
Adjuplex	Sigma Aldrich	Wegmann et al., 2015
p-nitrophenyl phosphate substrate	Sigma Aldrich	cat# 4264-83-9
alkaline phosphatase conjugated Streptavidin	Insight Biotechnologies	cat# 29071

**Deposited Data**		

Atomic coordinates, RVFV Gn- RV-Gn1 complex structure	Protein Data Bank	PDB: 6I9I

**Experimental Models: Cell Lines**		

HEK293T	ATCC	cat# CRL-1573
Vero	ECACC	cat# 8411301
Hybridoma RV-Gn1-2	This paper	N/A
HEK293F	Thermofisher	cat# R79007

**Experimental Models: Organisms/Strains**		

Male New Zealand white rabbit	Western Oregon	N/A
Female BALB/c mice	Envigo	N/A

**Oligonucleotides**		

Rabbit primer set	McCoy et al., 2016	See Table S4

**Recombinant DNA**		

RVFV M segment	GeneArt (Life Technologies)	accession # P21401
SFTSV M segment	GeneArt (Life Technologies)	accession # R4V2Q5
pHLSec Vector	Aricescu et al., 2006	N/A

**Software and Algorithms**		

Prism 5	GraphPad	www.graphpad.com
Coot	Emsley and Cowtan, 2004	N/A
Refmac5	Murshudov et al., 2011	N/A
Geneious v 8.1.3	http://www.geneious.com, (Kearse et al., 2012)	N/A
TempEst	Rambaut et al., 2016	N/A
BEAST v1.8.4	Drummond et al., 2012	N/A
MolProbity	Chen et al., 2010	N/A
PHASER	McCoy et al., 2007	N/A
XIA2	Winter, 2010	N/A
IMGT	Lefranc et al., 2009	N/A

**Other**		

Superdex 200 Increase 10/300 GL column	GE Healthcare Sciences	cat# 28990944

### Contact for Reagent and Resource Sharing

Further information and requests for resources and reagents should be directed to and will be fulfilled by the Lead Contact, Thomas A. Bowden (Thomas.Bowden@strubi.ox.ac.uk).

### Experimental Model and Subject Details

#### Rabbits

The rabbit immunization study was approved and carried out in accordance with protocols provided to the Institutional Animal Care and Use Committee (IACUC) at The Scripps Research Institute (TSRI; La Jolla, CA) under approval number #07-0021. The rabbits were kept, immunized, and bled at TSRI in compliance with the Animal Welfare Act and other federal statutes and regulations relating to animals, and in adherence to the *Guide for the Care and Use of Laboratory Animals* (National Research Council, 2011). Four male 8–10 week old New Zealand White rabbits were used in immunization studies.

#### Mice

All murine procedures with animals were undertaken according to the United Kingdom Animals (Scientific Procedures) Act 1986. These studies were approved by the ethical review process of Public Health England, Porton Down, UK, and by the Home Office, UK via Establishment License 70/1707 and project license P82D9CB4B. A set of humane end points based on clinical manifestation of disease were defined in the protocol of the project license. Female BALB/c 6-8 week old mice were used in these experiments.

#### Cell lines

HEK293F female embryonic kidney cells were cultured in Freestyle 293F expression media (GIBCO, Thermofisher). HEK293T female human embryonic kidney cells were cultured in DMEM supplemented with 10% FCS, non-essential amino acids and L-glutamine. (GIBCO, Thermofisher). Female vero cells were cultured in DMEM with 10% FCS. Hybridoma cell lines were generated commercially by Epitomics. Hybridoma cell cultures were grown in hybridoma SF media (Life Technologies). Cell lines were maintained in a humidified incubator at 37°C, supplied with 5%–8% CO_2_. HEK293F cells were agitated at 135 rpm. Cell lines were not authenticated following purchase.

### Method Details

#### Immunization of rabbits with recombinant RVFV Gn

Four male 8–10 week old New Zealand White rabbits were primed (intramuscularly) with the full-length RVFV Gn ectodomain (120 μg) adjuvanted with Adjuplex™ (Sigma Aldrich) (Wegmann et al., 2015) at a ratio of 1:5 adjuvant to immunogen in sterile PBS (1 mL total volume). Following immunization, a further two boosts were conducted at four week intervals. The final boost for rabbit 8315 was at week 16 and was performed intravenously seven days before Ab isolation. Sera were prepared from blood collected prior to immunization and seven days following each immunization/boost.

#### Murine RVFV infection model

Female BALB/c 6-8 week old mice were housed in groups of three. Groups of six mice (randomly assigned, two boxes per group) were treated intravenously with RV-Gn1 (200 μg or 10 μg) or received no treatment. An additional control group of five mice was treated with 200 μg of a non-RVFV rabbit mAb control. Four to six hours post-treatment, mice were challenged subcutaneously with 20 pfu of RVFV, strain ZH501. Mice were monitored six times daily for symptoms of infection and were culled when the determined humane endpoint was reached. Mouse survival was analyzed using a Kaplan-Meier test with log-rank using GraphPad Prism.

#### mAb isolation by hybridoma fusion

Seven days following the final boost for rabbit 8315, a spleenectomy was performed and hybridoma cell lines were generated and then selected by screening the cell supernatant for RVFV neutralization potency (Epitomics). Hybridoma cell cultures were grown for 7–10 days. mAbs were purified from cell supernatant using a Protein G column and buffer exchanged to 10 mM Tris pH 8.0 150 mM NaCl.

#### Phleboviral Gn expression

The cDNA of the RVFV Gn glycoprotein (UniProt accession number P21401) was synthetized by GeneArt (Life Technologies). Two RVFV Gn constructs were cloned into the pHLSec mammalian expression vector (Aricescu et al., 2006): the ectodomain construct for immunization (residues 192–560) and a short crystallization construct representing the residues observed in our previously reported structure of RVFV Gn (residues 168–483) (Halldorsson et al., 2018). The SFTSV Gn glycoprotein (UniProt accession number R4V2Q5) residues 20–341 was synthetized and subcloned as described above. Proteins were expressed in transiently transfected (HEK) 293T cells (ATCC CRL-1573). Cell supernatants were harvested four days following transfection, and purified by immobilized nickel-affinity chromatography (5 mL HisTrap FF crude column and ÄKTA FPLC system, GE Healthcare) followed by size exclusion chromatography (SEC).

#### Antigen-specific B cell sorting

PBMCs were purified from rabbit 8315 seven days after the third boost using a Lymphoprep (STEMCELL Technology) density gradient. PBMCs were cryopreserved in FBS plus 10% DMSO. Fluorescence-activated cell sorting of cryopreserved PBMCs was performed. PBMCs were stained with anti-CD3-FITC (Santa Cruz Biotechnology), anti-IgM-PE (Southern Biotech), anti-IgG-PerCP-Cy5.5 (Santa Cruz Biotechnology) and hexahistidine-tagged RVFV-Gn. Cells were washed and anti-HIS-APC (Abcam) was added. CD3^-^IgM^-^IgG^+^RVFV Gn^+^ cells were sorted into individual wells containing RNase OUT (Invitrogen), First Strand SuperScript III buffer, DTT and H_2_O (Invitrogen) and RNA was converted into cDNA (SuperScript III Reverse Transcriptase, Invitrogen) using random hexamers following the manufacturer’s protocol.

#### Full-length Ab cloning and expression

The rabbit Ab variable regions of heavy and kappa chains were PCR amplified using previously described primers and PCR conditions (Table S4) (McCoy et al., 2016). PCR products were purified and cloned into an expression plasmid adapted from the pFUSE-rIgG-Fc and pFUSE2-CLIg-rK1 vectors (InvivoGen) using the Gibson Assembly® Master Mix (NEB) under ampicillin selection following the manufacturer’s protocol. Ab variable regions were sequenced by Sanger sequencing.

Ab heavy and light plasmids generated through B cell sorting were co-transfected at a 1:1 ratio into HEK293F cells (Thermofisher) using PEI Max 40K (linear polyethylenimine hydrochloride, Polysciences, Inc.). Ab supernatants were harvested four days following transfection and purified using protein G affinity chromatography following the maunfacturers protocol (GE Healthcare).

#### Fab cloning and expression

Fab fragments were cloned from hybridoma cells, using the RNA extraction (RNAeasy, QIAGEN) and RT-PCR strategy described above. Isolated DNA for Fab fragments was then cloned into the pHLsec vector. A C-terminal His_6_-tag was included in the heavy chain construct and both chains were co-expressed (1:1 (w/w) ratio of Fab heavy to light chain expressing plasmids) in HEK293T cells and purified by immobilized nickel-affinity chromatography (5 mL HisTrap FF crude column and ÄKTA FPLC system, GE Healthcare) followed by SEC into a buffer containing 10 mM TRIS pH 8.0, 150 mM NaCl.

#### Ab binding experiments

High binding ELISA 96 half-well microplates (Corning) were coated with purified RVFV Gn (25 μL, 3 μg/mL in PBS) overnight at 4°C. Plates were washed five times with PBS containing 0.05% Tween20 (PBS-T) and blocked with blocking buffer (5% non-fat milk in PBS-T) for 1 h at RT. The blocking buffer was removed and serial diluted Ab (starting at 50 μg/mL, 1:5 dilution in blocking buffer) was added for 2 h at RT. Plates were washed five times with PBS-T. Secondary Ab (goat anti-rabbit IgG F(ab’)_2_, AP conjugate, Invitrogen, 1:1000) was added for 1 h and plates were washed, as described above. The p-nitrophenyl phosphate substrate (Sigma) was added to detect binding and the ODs were measured at 405 nm.

An ELISA to determine cross-reactivity between RV-Gn1 and SFTSV Gn was also performed. The ELISA was performed as above: plates were coated with either RVFV Gn or SFTSV Gn, serially diluted RV-Gn1 (starting at 10μg/ml, 1:5 dilution in blocking buffer) was used for primary detection, and goat anti-rabbit was used for secondary detection. The Pierce TMB substrate kit (ThermoFisher) was added to detect binding and OD were measured at 450 nm.

#### Fab fragment competition ELISA

ELISA plates were coated and blocked as above. Serial diluted Fab fragments were added (starting at 100 μg/ml, 1:5 dilution in blocking buffer) for 30 min, and then equal volumes of the competing Abs were added at a constant concentration (twice the IC80 RVFV Gn binding concentration) for 1.5 h at RT. Biotinylated Fc specific rabbit cross-reactive secondary Ab (goat anti-mouse IgG Fc, biotin conjugate, Invitrogen, 1:200) was added for 30 min. The plates were washed as above and alkaline phosphatase conjugated Streptavidin (Insight Biotechnologies, 1:1000) was added for 30 min. Plates were washed and competition detected as described above.

#### Plaque reduction neutralization test

100 μL of three-fold serial-diluted Mab or control Ab (anti-HIV monoclonal Ab PG9 (Walker et al., 2011, Walker et al., 2009)) was mixed with an equal volume of 100 plaque forming units (pfu.) RVFV ZH501 at 37°C for 1h. The virus–Ab mix was then transferred to 80–90% confluent Vero cells in a 24-well plate and incubated at 37°C for 1 h. After incubation liquid overlay, MEM with (1% avicel, 10% FBS, 1% antibiotic/antimycotic solution) was added. The plates were then incubated for 3–4 days, and fixed and stained, as described above. Plaques were then counted for each well and the neutralization percentage was calculated relative to the corresponding PG9 Mab control. IC_50_ values were calculated in GraphPad Prism using a least-squares non-linear fit dose-response curve.

#### Crystallization and structure determination

RVFV Gn and RV-Gn1 were complexed and purified by SEC using a Superdex 200 10/300 Increase column (GE Healthcare). The RVFV Gn–RV-Gn1 complex was crystallized using the sitting drop vapor diffusion method (Walter et al., 2005) after 185 days at RT at a concentration of 9.5 mg mL^−1^, by mixing 100 nL of protein in 10 mM TRIS pH 8.0, 150 mM NaCl buffer and 100 nL 20% w/v PEG 500, 0.1 M bis-Tris pH 6.5. Crystals were cryo-cooled in the precipitant containing 25% glycerol.

X-ray data were recorded at Beamline I03 at Diamond Light Source (Didcot, UK) on a Pilatus 6MF detector (Dectris). X-ray data were indexed, integrated, and scaled with XIA2 (Winter, 2010). The structure of RVFV Gn–RV-Gn1 was phased by molecular replacement with PHASER (McCoy et al., 2007) using the crystal structures of a rabbit Fab fragment (PDB:4J02) as a search model. Iterative model building was performed with COOT (Emsley and Cowtan, 2004). Structure refinement was performed with Refmac5 (Murshudov et al., 2011) in the CCP4 suite. The final refined structure was validated with MolProbity (Chen et al., 2010).

### Quantification and Statistical Analysis

#### Phylogenetic and molecular evolution analysis

Publicly available sequences encoding the full-length RVFV M segment (∼3600 bp) with known sample dates were obtained from GenBank and manually aligned. The final sequence alignment comprised 98 sequences, sampled from 1951 to 2010. An initial neighbor-joining tree was constructed in Geneious v 8.1.3 (http://www.geneious.com, (Kearse et al., 2012) using a HKY nucleotide substitution model and 100 bootstrap replicates. The presence of a sufficient temporal signal in the alignment for molecular clock analysis was confirmed using TempEst (Rambaut et al., 2016). For the dN/dS analysis, Bayesian molecular clock phylogenies were estimated using BEAST v1.8.4 (Drummond et al., 2012). We used a log-normal relaxed molecular clock model (Drummond et al., 2006), a Bayesian Skygrid coalescent prior (Gill et al., 2013), and a codon-structured nucleotide substitution model (Shapiro et al., 2006). Two independent MCMC runs of 50 million steps were computed to ensure that stationarity and convergence had been achieved. An empirical distribution of 9,000 molecular clock phylogenies was obtained by combining (after the removal of burnin) the posterior tree distributions of each run. This empirical distribution was used subsequently to estimate dN/dS ratios using the renaissance counting method (Lemey et al., 2012) implemented in BEAST v1.8.4. The alignment was partitioned into RVFV Gn and Gc glycoproteins, and the RVFV Gn was subdivided further into domains A, B, and the β-ribbon domain (β). Hierarchical priors were applied to the substitution model parameters for each partition, which enabled statistical strength for individual parameters to be shared across different partitions (Suchard et al., 2003). Two independent MCMC runs of 10 million steps were computed for this analysis using BEAST version 1.8.4.

### Data and Software Availability

Atomic coordinates and structure factors of the RVFV Gn-RV-Gn1 complex have been deposited in the PDB (accession code 6I9I).

## Supplemental Information

Document S1. Figures S1–S3 and Tables S1–S4

Document S2. Article plus Supplemental Information
