# Supplementary material for: A Protective Monoclonal Antibody Targets a Site of Vulnerability on the Surface of Rift Valley Fever Virus
Source: Cell Rep. 2018 Dec 26;25(13):3750–3758.e4. doi: 10.1016/j.celrep.2018.12.001 (PMC6315105; doi:10.1016/j.celrep.2018.12.001)
Supplement: Document S1. Figures S1–S3 and Tables S1–S4 [file mmc1.pdf]

Cell Reports, Volume *25*

Supplemental Information

A Protective Monoclonal Antibody Targets a Site of Vulnerability on the Surface of Rift Valley Fever Virus

Elizabeth R. Allen, Stefanie A. Krumm, Jayna Raghwani, Steinar Halldorsson, Angela Elliott, Victoria A. Graham, Elina Koudriakova, Karl Harlos, Daniel Wright, George M. Warimwe, Benjamin Brennan, Juha T. Huiskonen, Stuart D. Dowall, Richard M. Elliott, Oliver G. Pybus, Dennis R. Burton, Roger Hewson, Katie J. Doores, and Thomas A. Bowden

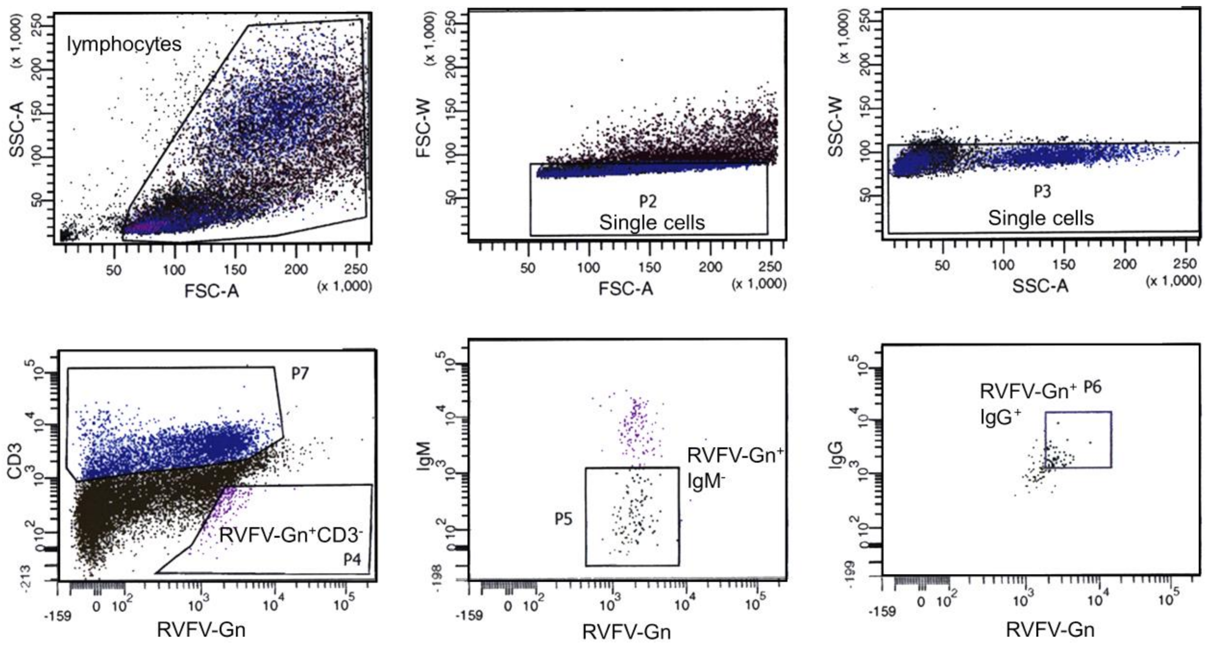

Supplementary Fig. S1. (Related to Fig. 1). Antigen-specific fluorescence-activated cell sorting gating strategy. $CD3^{-}IgM^{-}IgG^{+}RVFV\ Gn^{+}$ PBMCs were sorted into single wells of a 96-well plate for subsequent sequence analysis.

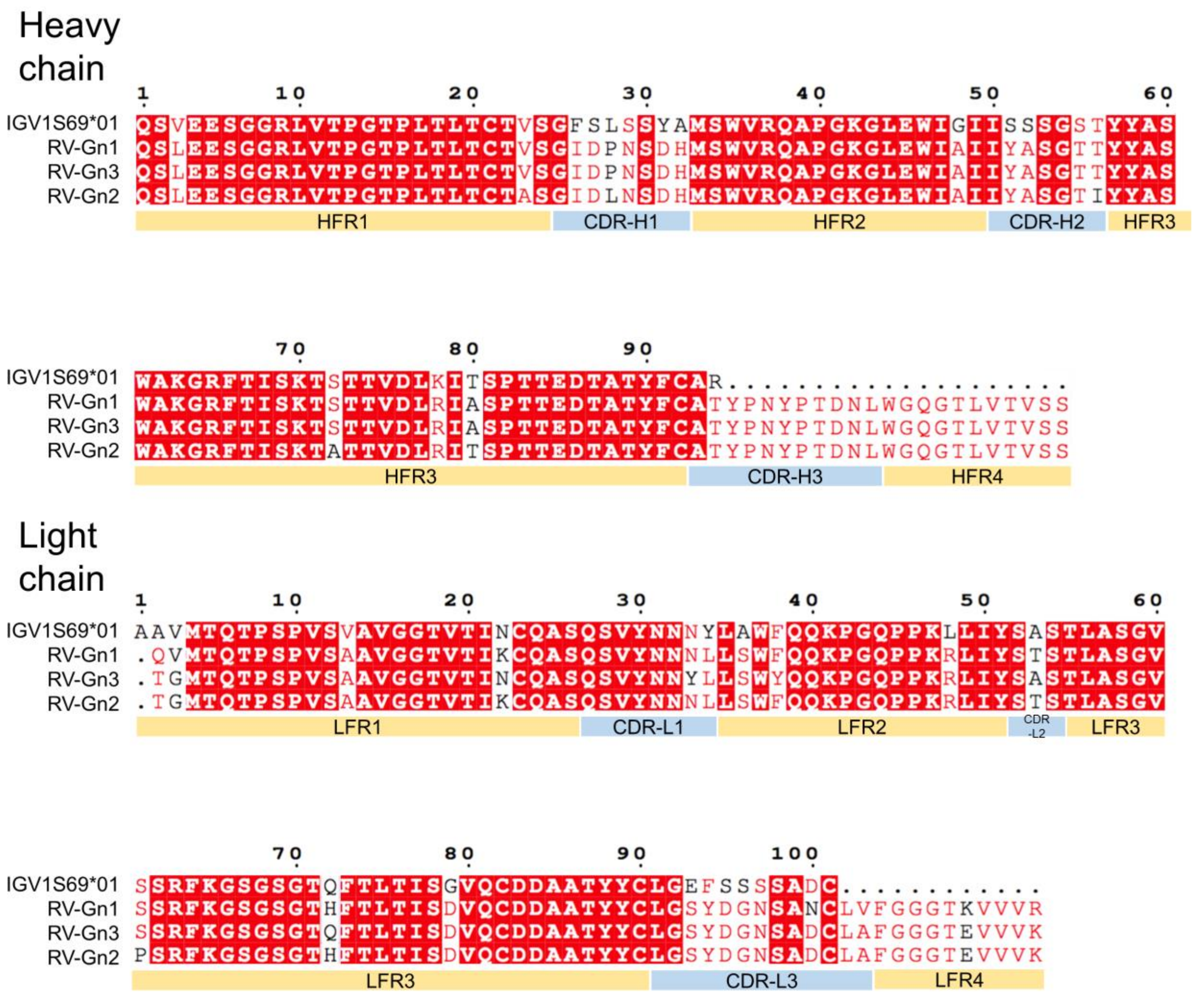

Supplementary Fig. S2. (Related to Fig. 2.) Amino acid sequence comparison of three isolated antibodies (RV-Gn1–3) and their corresponding germline sequence. CDR regions are shown. Top germline V-gene hits were determined using the international immunogenetics information system (IMGT) database (Lefranc et al., 1999). The sequence alignment was determined by Clustal Omega (Sievers et al., 2011) and plotted with ESPRIPT (Robert and Gouet, 2014).

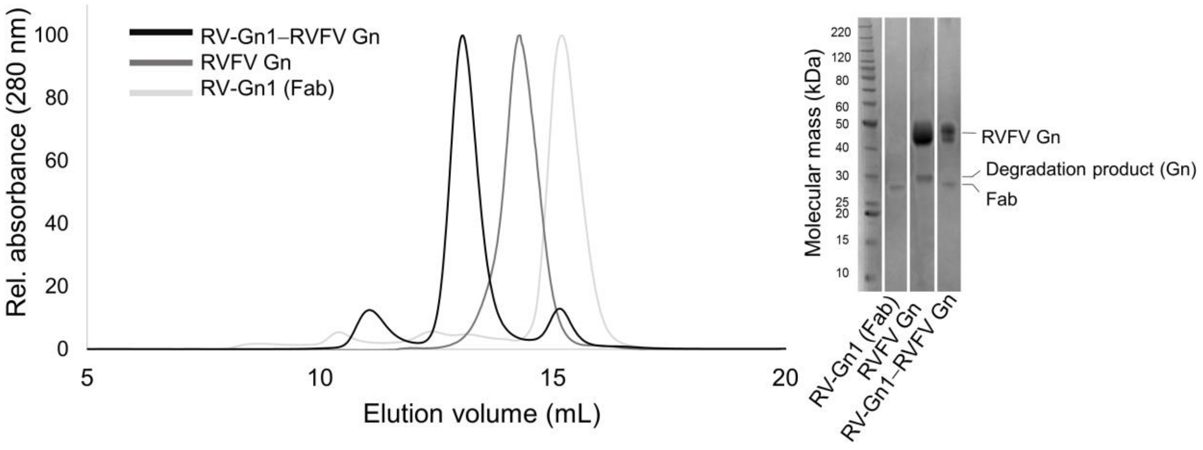

Supplementary Fig. S3. (Related to Fig. 3.) Complexation of RVFV-Gn with Fab RV-Gn1. (left) Samples were run on a Superdex 200 10/30 column (Amersham) equilibrated with 150 mM NaCl and 10 mM Tris-HCl pH 8.0. (right) SDS-PAGE analysis of RVFV Gn and the RV-Gn1 Fab fragment prior to co-crystallization.

Supplementary Table S1. Related to Fig. 1. Plaque reduction assay of sera from immunized rabbits with RVFV Gn.

	**Serum dilution resulting in a 50% plaque reduction**	
**Animal ID**	**Bleed 2**	**Bleed 3**
8312	1:256	1:4096
8313	1:2048	1:2048
8314	1:512	1:4096
8315	>1:4096	1:8192

Supplementary Table S2. Related to Fig. 2. Analysis of isolated mAb sequences and their germlines. Antibody germline V, D, and J genes were assigned using the international immunogenetics information system (IMGT) database (Lefranc et al., 1999).

Heavy	V region	Identity (% nt)	J region	Identity (% nt)	D region	CDR1	CDR2	CDR3
RV-Gn1	1S69*01	91.7	4*01	83.3	7-1*01	GIDPNSDH	IYASGTT	CATYPNYPTDNLW
RV-Gn2	1S69*01	91.3	4*01	81.3	7-1*01	GIDLNSDH	IYASGTI	CATYPNYPTDNLW
RV-Gn3	1S69*01	89.9	4*01	72.9	7-1*01	GIDPNSDH	IYASGTT	CATYPNYPTDNLW

Kappa	V region	Identity (% nt)	J region	Identity (% nt)	D region	CDR2	CDR3
RV-Gn1	1S18*01	91.1	1-2*01	90.0	QSVYNNYL	SAS	CLGSYDGNSADCLAF
RV-Gn2	1S18*01	92.2	1-2*01	92.1	QSVYNNNL	STS	CLGSYDGNSADCLAF
RV-Gn3	1S18*01	92.2	1-2*01	73.7	QSVYNNNL	STS	CLGSYDGNSANCLVF

Supplementary Table S3. Related to Fig.3 Crystallographic data and refinement statistics for RVFV Gn–RV-Gn1.

	RVFV Gn–RV-Gn1
**Data collection statistics**	
Beamline	Diamond i03
Space group	*P*1
Cell dimensions	
a, *b*, *c* (Å)	51.6, 62.0, 78.9
α, β, γ (°)	81.0, 77.5, 84.7
Resolution range (Å)	23.6–1.98 (2.03–1.98)[a]
R_{merge}	0.072 (0.651)
I/σ I	5.8 (1.1)
$CC_{1/2}$	0.98 (0.67)
Completeness (%)	96.2 (95.3)
Redundancy	1.8 (1.7)
**Refinement statistics**	
Resolution (Å)	23.6–1.98 (2.03–1.98)
No. reflections	59,882
R_{work} / R_{free}[b] (%)	20.3/24.1
No. atoms	
Protein	7,049
Ligand/ion	n/a
Water	433
B-factors	
Protein	48.4
Ligand/ion	n/a
Water	49.1
R.m.s. deviations[c]	
Bond lengths(Å)	0.006
Bond angles (°)	1.1
Ramachandran analysis[d]	
Residues in favored region (%)	98.1
Residues in allowed region (%)	1.9

[a]Numbers in parentheses refer to the relevant outer resolution shell.
[b]R_{free} is calculated as for R_{work}, but using only 5% of the data which were sequestered prior to refinement.
[c]r.m.s. deviations: root mean square deviation from ideal geometry.
[d]Determined using the MolProbity server (Chen et al., 2010).

Supplementary Table 4. Related to Methods. Primers used for mAb isolation from rabbits.

**First PCR IgH Forward**	
RHFout	ATGGAGACTGGGCTGCGCTGGCTTC
**Reverse**	
RHRout1	GTCCTTGGGTTTTGGGGGAAAGATGAA
RHRout2	GTCCCCGCAGCAGGGGGCCAGTGGGAA
RHRout3	CTCCTCCCGGGGAGGGCCCATGGTGTA
**Second PCR IgH Forward**	
HC FW1-1 f	TGCACTAAGTCTTGCACTTGTCACGAATTCGCAGAGCCTGGAAGAGTCTGGCGG
HC FW1-2 f	TGCACTAAGTCTTGCACTTGTCACGAATTCGCAGAGCCTGGAGGAGTCCGGGGG
HC FW1-3 f	TGCACTAAGTCTTGCACTTGTCACGAATTCGCAGAGCCTGGAGCAGTCCGGAGG
HC FW1-4 f	TGCACTAAGTCTTGCACTTGTCACGAATTCGCAGAGCCTGGTGGAGTCCGGGGG
HC FW1-5 f	TGCACTAAGTCTTGCACTTGTCACGAATTCGCAGAGCCTGGTGGAGTCCGGAGG
**Reverse**	
HC FW4-1 r	ATGGAGCCTTAGGTTGCCCACTCGAG ACGATCACGAGGGTGCC
HC FW4-2 r	ATGGAGCCTTAGGTTGCCCACTCGAG ACGGTGACCAGGGTGCC
HC FW4-3 r	ATGGAGCCTTAGGTTGCCCACTCGAG ACGGTAACCAGGGTGCC
HC FW4-4 r	ATGGAGCCTTAGGTTGCCCACTCGAG ACGCTCACCACGCTGCT
R56HR Xho	ATGGAGCCTTAGGTTGCCCACTCGAGACGGTGACCAGGGTG
**First PCR IgK Forward**	
RVK1	GCGCCGGAGCTCGTGATGACCCAGACTCCA
RVK2	GCGCCGGAGCTCGATATGACCCAGACTCCA
**Reverse**	
RCK1	GCGCCGTCTAGACTAACAGTCACCCCTATTGAAGC
RCK2	GCGCCGTCTAGACTAACAGTTCTTCCTACTGAAGC
IGκ	GATGCCAGTTGTTTGGGTGGT
**Second PCR IgK Forward**	
RbKappaF2	TGCACTAAGTCTTGCACTTGTCACGAATTCGGACATCGT
LC FW1-1 f	TGCACTAAGTCTTGCACTTGTCACGAATTCGGACCAGGTGCTGACCCAGACTCC
LC FW1-2 f	TGCACTAAGTCTTGCACTTGTCACGAATTCGGACCAGGTGCTGACCCAAACACC
LC FW1-3 f	TGCACTAAGTCTTGCACTTGTCACGAATTCGGACCAGGTGCTGACCCAGACTGC
LC FW1-4 f	TGCACTAAGTCTTGCACTTGTCACGAATTCGGACCAGGTGATGACCCAGACACC
LC FW1-5 f	TGCACTAAGTCTTGCACTTGTCACGAATTCGGACCAGGTGATGACCCAGACTCC

**Reverse**	
RkRBamhI	GGAGGACAGAAGGCGCAACTGGATCACCTTTGACC
LC FW4-1 r	GGAGGACAGAAGGCGCAACTGGATCCCCCCGCACGACCACCTCTGTTC
LC FW4-2 r	GGAGGACAGAAGGCGCAACTGGATCCCCCCGCACGACCACCTCGGTCC
LC FW4-3 r	GGAGGACAGAAGGCGCAACTGGATCCCCCCGCACGACCACCTTGGTCC
